# Surgical and functional outcomes of pediatric pancreatic solid pseudopapillary neoplasm: A retrospective study

**DOI:** 10.1371/journal.pone.0357019

**Published:** 2026-09-10

**Authors:** Hoan Manh Vu, Hien Duy Pham, Son Cong Nguyen, Nhi Yen Bui, Hoang Viet Nguyen, Ngoc Luong Khanh Nguyen, Quang Thanh Nguyen

**Affiliations:** 1 College of Health Sciences, VinUniversity, Hanoi, Vietnam; 2 Department of Pediatric Surgery, Vietnam National Children’s Hospital, Hanoi, Vietnam; 3 The Royal College of Surgeons in Ireland and University College Dublin Malaysia Campus, George Town, Penang, Malaysia; 4 Innovations in Health Sciences, VinUniversity, Hanoi, Vietnam; Sichuan University, CHINA

## Abstract

**Background:**

Pediatric pancreatic solid pseudopapillary neoplasm (SPN) is rare, but it represents a clinically important pancreatic tumor in children. The optimal operative strategy remains uncertain, particularly the balance between tumor clearance, postoperative morbidity, and preservation of pancreatic function. This study evaluated surgical, oncologic, and functional outcomes after a selective strategy incorporating parenchyma-preserving procedures.

**Methods:**

A retrospective cohort study included patients aged ≤16 years who underwent surgery for pathologically confirmed SPN at a tertiary hospital from January 2021 to December 2024. Clinical, imaging, operative, pathologic, postoperative, recurrence, and pancreatic functional outcomes were reviewed.

**Results:**

Thirty-two patients were included; 28 were female (87.5%), and the mean age was 11.1 ± 2.1 years. Abdominal pain was the most common presentation (81.3%). The median tumor diameter was 42.5 mm (range, 19–120). Tumors were located in the pancreatic head in 15 patients (46.8%) and the body/tail in 17 (53.2%). Parenchyma-preserving procedures were performed in 19 patients (59.4%). Distal pancreatectomy was performed in 12 patients, with spleen preservation in 9. Early complications occurred in 5 patients, all with pancreatic head tumors, including one grade C pancreatic fistula that required reoperation. There was no mortality. R0 resection was achieved in 21 patients (65.6%). During a mean follow-up of 34.4 ± 9.8 months, one local recurrence occurred after R1 pancreatic head enucleation and was treated with pancreaticoduodenectomy. No clinically recognized endocrine insufficiency developed. Transient exocrine insufficiency occurred in 11 of 26 assessed patients and resolved in all. Exploratory subgroup analyses were underpowered and did not establish equivalent outcomes between operative strategies or margin groups.

**Conclusions:**

Parenchyma-preserving surgery was feasible in selected children with SPN, but pancreatic head enucleation carried fistula and margin-related concerns. These findings support selective rather than routine pancreatic preservation, with structured long-term surveillance.

## Introduction

Solid pseudopapillary neoplasm (SPN) is a rare pancreatic tumor with low-grade malignant potential, accounting for approximately 0.9%–2.7% of exocrine pancreatic neoplasms and about 5% of cystic pancreatic tumors [1]. Despite an estimated annual incidence of only 0.1–0.2 per million children, SPN is one of the most frequent pancreatic tumors with malignant potential in the pediatric population [2–4]. It predominantly affects adolescent girls and commonly presents with nonspecific abdominal pain or as an incidental mass [2,5]. Because pediatric pancreatic tumors are uncommon and SPN may contain variable solid and cystic components, preoperative diagnosis and operative planning can remain challenging.

Complete surgical resection is the standard treatment, and recurrence is generally reported in 2%–10% of patients [1]. In children, however, long life expectancy makes preservation of pancreatic endocrine and exocrine function an important additional objective. Conventional resections, including pancreaticoduodenectomy and distal pancreatectomy, provide reliable tumor clearance but may remove substantial normal pancreatic parenchyma [3,6]. Consequently, enucleation, central pancreatectomy, and spleen-preserving distal pancreatectomy have increasingly been used in selected patients [3,7,8].

The benefits of parenchyma preservation must be balanced against perioperative and oncologic risks. These procedures may increase postoperative pancreatic fistula and may result in close or microscopically positive margins, particularly for tumors in the pancreatic head or near the main pancreatic duct and bile duct [1,3,7–9]. Their long-term functional advantage and the oncologic significance of R1 resection remain uncertain because pediatric cohorts are small, recurrence events are uncommon, and endocrine and exocrine outcomes are inconsistently assessed [1–3,6]. Two previous Vietnamese-language publications from our institution described an earlier 15-patient series and short-term outcomes after pancreatic head-preserving surgery [10,11]. The present study evaluates the full consecutive 2021–2024 cohort and extends the analysis to operative selection, postoperative morbidity, pathology, margin status, recurrence, and pancreatic endocrine and exocrine outcomes. We aimed to describe these outcomes and to examine the trade-off between pancreatic preservation, perioperative risk, and tumor control.

## Methods

### Study design and settings

This single-center retrospective cohort included consecutive patients aged 16 years or younger who underwent resection of pathologically confirmed pancreatic SPN at Vietnam National Children’s Hospital from January 1, 2021, through December 31, 2024. Follow-up was updated through December 31, 2025. Cases were identified from the pathology registry and verified against operative and hospital records. Patients with recurrent SPN at presentation, biopsy without therapeutic resection, previous pancreatic surgery elsewhere, incomplete records, severe comorbidity, or another malignancy affecting outcome assessment were excluded. Because pediatric SPN is rare, no formal sample size calculation was performed; all consecutive eligible patients were included. The study was reported in accordance with the Strengthening the Reporting of Observational Studies in Epidemiology statement [12].

Medical records were accessed for research purposes from October 1, 2025, through February 28, 2026. Investigators had access to identifiable information during data extraction; the analytic dataset was subsequently anonymized, and identifying information was not retained for analysis. The Biomedical Research Ethics Committee of Vietnam National Children’s Hospital approved the study (Decision No. 701/BVNTW-HĐĐĐ; IRB-VN01037/IRB00011976/FWA00028418). The study was conducted in accordance with the Declaration of Helsinki and relevant institutional regulations. The ethics committee waived the requirement for informed consent because of the retrospective design and use of anonymized data.

### Preoperative assessment and operative strategy

All patients underwent contrast-enhanced CT or MRI. Tumor size was defined as the largest imaging diameter, and location was categorized as pancreatic head or body/tail. Preoperative biopsy was not routinely performed for a resectable lesion with imaging typical of SPN because tissue confirmation was unlikely to alter the planned resection and sampling may be nondiagnostic; biopsy was reserved for atypical or unresectable lesions or when histology would change management [1].

All 32 operations were performed through an open approach; no laparoscopic or robotic procedures were performed. The procedure was selected according to tumor location, proximity to the main pancreatic duct and bile duct, adjacent-organ or vascular involvement, and intraoperative resectability. Enucleation was considered for well-circumscribed pancreatic head lesions that were separable from the main pancreatic duct, bile duct, duodenum, and major vessels. Pancreaticoduodenectomy was reserved for lesions that could not be safely separated. Central pancreatectomy was considered for neck or body lesions, and distal pancreatectomy was used for body or tail lesions. All nine spleen-preserving distal pancreatectomies used splenic-vessel preservation according to the Kimura technique; no Warshaw procedure was performed. Frozen section and resection of suspicious metastatic tissue were used when intraoperative findings required diagnostic or oncologic clarification.

### Pathology, outcomes, and follow-up

All resection specimens were reviewed by institutional pathologists. Hematoxylin and eosin morphology established the diagnosis when characteristic; immunohistochemistry was used when morphology was equivocal or pancreatic neuroendocrine tumor or another lesion was considered. Tumors were classified according to the World Health Organization classification of digestive system tumors [13]. Margin status was extracted from final pathology reports and categorized as R0, R1, or R2 according to the residual tumor classification. Margin sampling and reporting reflected routine institutional practice and were not prospectively standardized.

The primary outcomes were early postoperative morbidity and tumor recurrence. Early complications occurred during the index admission or within 30 days and were classified according to Clavien-Dindo [14]. Clinically relevant POPF was defined as grade B or C according to the International Study Group of Pancreatic Surgery [15]. Bile leak and delayed gastric emptying were defined using International Study Group criteria [16,17]. Secondary outcomes included operative time, intraoperative events, reoperation, length of stay, margin status, survival, and pancreatic endocrine and exocrine outcomes.

Follow-up was based on pediatric surgical outpatient visits and hospital-record review and was not governed by a uniform prospective schedule. The minimum follow-up was 12 months. Recorded outcomes included symptoms, recurrence, survival, fecal elastase when available, and clinically recognized postoperative diabetes. PERT, including Creon, serial height, weight, or body mass index trajectories, protocolized fasting glucose or HbA1c testing, and hepatic steatosis could not be uniformly extracted and were not analyzed. Missing data were handled by available-case analysis, and outcome-specific denominators are reported.

### Statistical analysis

Categorical variables are presented as n (%) and continuous variables as mean ± standard deviation or median (range), according to distribution. For exploratory comparisons, enucleation and central pancreatectomy were classified as parenchyma-preserving procedures, whereas distal pancreatectomy and pancreaticoduodenectomy were classified as conventional resections. Comparisons used Student’s t test, the Mann–Whitney U test, the chi-square test, or Fisher’s exact test, as appropriate. Exploratory analyses evaluated clinically relevant POPF and recurrence by parenchyma-preserving versus conventional resection and enucleation versus other procedures, as well as recurrence by R1 versus R0 margin status. These analyses were considered hypothesis-generating because of the small cohort and low event counts. All tests were two-sided, and p < 0.05 was considered statistically significant. Analyses were performed using SPSS version 26.0 (IBM Corp., Armonk, NY, USA).

## Results

### Patient characteristics and imaging

All 32 eligible patients were included. There were 28 girls (87.5%) and 4 boys, with a mean age of 11.1 ± 2.1 years (range, 7–15). Abdominal pain was the most common presentation (26/32, 81.3%). The median tumor diameter was 42.5 mm (range, 19–120); 15 tumors were located in the pancreatic head and 17 in the body/tail. Mixed solid-cystic morphology was present in 20/32 patients (62.5%), and calcification was present in 8/32 (25.0%). Preoperative imaging suggested SPN in 28 patients, whereas 4 lesions were interpreted as pancreatic pseudocysts (Table 1).

**Table 1:** Baseline clinical and imaging characteristics.

Characteristic	Value
Number of patients	32
Age, years	11.1 ± 2.1 (range, 7–15)
**Sex**	
Female	28 (87.5%)
Male	4 (12.5%)
**Primary presentation**	
Abdominal pain	26 (81.3%)
Digestive symptoms	2 (6.2%)
Incidental finding	2 (6.2%)
Detected after abdominal trauma	1 (3.1%)
Palpable abdominal mass	1 (3.1%)
**Preoperative imaging**	
CT	28 (87.5%)
MRI	4 (12.5%)
**Preoperative diagnosis**	
SPN	28 (87.5%)
Pancreatic pseudocyst	4 (12.5%)
**Tumor size, mm**	
Median diameter	42.5 (range, 19–120)
≤50	21 (65.6%)
51-100	8 (25.0%)
>100	3 (9.4%)
**Tumor location**	
Pancreatic head	15 (46.8%)
Pancreatic body/tail	17 (53.2%)
**Tumor morphology**	
Solid	11 (34.4%)
Cystic	1 (3.1%)
Mixed solid-cystic	20 (62.5%)
Calcification	8 (25.0%)
**Associated imaging findings**	
Biliary dilatation	2 (6.3%)
Main pancreatic duct dilatation	4 (12.5%)
Regional lymphadenopathy	1 (3.1%)

*Values are n (%) unless otherwise stated. CT, computed tomography; MRI, magnetic resonance imaging; SPN, solid pseudopapillary neoplasm.*

### Operative management

All procedures were open. Among 15 pancreatic head tumors, 14 underwent enucleation and 1 underwent pancreaticoduodenectomy because the lesion could not be safely separated from the duodenum. Among 17 body/tail tumors, 5 underwent central pancreatectomy and 12 underwent distal pancreatectomy. Parenchyma-preserving procedures were performed in 19 patients (59.4%). Nine of 12 distal pancreatectomies preserved the spleen using the Kimura technique. Four patients undergoing pancreatic head enucleation also received a Roux-en-Y jejunal anastomosis to the operative bed because of concern for pancreatic leakage. One patient underwent resection of the primary tumor, omental metastasis, and a retropancreatic metastatic lymph node (Table 2).

**Table 2:** Operative management and intraoperative outcomes.

Variable	Value
**Surgical access**	
Open approach	32/32 (100%)
**Primary procedure**	
Enucleation of pancreatic head tumor	14 (43.7%)
Pancreaticoduodenectomy	1 (3.1%)
Central pancreatectomy	5 (15.7%)
Distal pancreatectomy	12 (37.5%)
**Additional procedure or organ preservation**	
Roux-en-Y jejunal anastomosis after head enucleation	4/14 (28.6%)
Spleen-preserving distal pancreatectomy, Kimura technique	9/12 (75.0%)
Distal pancreatectomy with splenectomy	3/12 (25.0%)
**Operative time, minutes**	
Enucleation	152.9 ± 57.9 (range, 86–272)
Pancreaticoduodenectomy	265
Central pancreatectomy	188.2 ± 46.4 (range, 160–270)
Distal pancreatectomy	137.8 ± 36.8 (range, 90–210)
**Intraoperative finding or event**	
Duodenal invasion requiring pancreaticoduodenectomy	1 (3.1%)
Omental and retropancreatic nodal metastases	1 (3.1%)
Splenic vessel injury requiring splenectomy	3 (9.4%)
Common bile duct injury, repaired intraoperatively	1 (3.1%)
Portal vein injury, repaired intraoperatively	1 (3.1%)
Intraoperative blood transfusion	2 (6.2%)

*Values are n (%), n/N (%), or mean ± standard deviation with range. All spleen-preserving distal pancreatectomies used splenic-vessel preservation (Kimura technique); no Warshaw procedure was performed.*

### Postoperative and pathologic outcomes

Any early postoperative complication occurred in 5 patients (15.6%), all after surgery for pancreatic head tumors, compared with none after body/tail surgery (Fisher exact p = 0.015). Complication categories overlapped; all 5 affected patients had DGE. Clinically relevant POPF occurred after pancreatic head enucleation in 2 patients: one grade B fistula resolved with conservative treatment, and one grade C fistula required pancreatic-enteric reconstruction. No postoperative bleeding, wound infection, early bowel obstruction, or perioperative death occurred. Median hospital stay was 10 days (range, 5–32), without a statistically significant difference by tumor location (p = 0.053).

Characteristic hematoxylin and eosin morphology was considered sufficient for diagnosis in 22 patients. Immunohistochemistry was performed in 10 patients for diagnostic confirmation or differential diagnosis, particularly to exclude pancreatic neuroendocrine tumor; chromogranin was negative in all tested cases. Ki-67 was assessed in 6 patients and was less than 10% in all. R0 resection was reported in 21 patients (65.6%) and R1 resection in 11 (34.4%); no R2 resection occurred (Table 3).

**Table 3:** Early postoperative outcomes and resection margins by tumor location.

Outcome	Head tumors (n = 15)	Body/tail tumors (n = 17)	Overall (n = 32)
Any early postoperative complication	5 (33.3%)	0	5 (15.6%)
Delayed gastric emptying	5 (33.3%)	0	5 (15.6%)
Acute pancreatitis	2 (13.3%)	0	2 (6.2%)
Grade B POPF	1 (6.7%)	0	1 (3.1%)
Grade C POPF requiring reoperation	1 (6.7%)	0	1 (3.1%)
Bile leak	1 (6.7%)	0	1 (3.1%)
**Resection margin**			
R0	9 (60.0%)	12 (70.6%)	21 (65.6%)
R1	6 (40.0%)	5 (29.4%)	11 (34.4%)

*Values are n (%). POPF, postoperative pancreatic fistula. Complication categories were not mutually exclusive; all five patients with an early complication also had delayed gastric emptying. Any early complication was more frequent after surgery for pancreatic head tumors than after body/tail surgery (Fisher exact p = 0.015).*

### Long-term outcomes and exploratory subgroup analyses

During a mean follow-up of 34.4 ± 9.8 months, one patient developed local recurrence 14 months after enucleation of a pancreatic head SPN with an R1 margin, perineural invasion, and a Ki-67 index of 5%. She underwent pancreaticoduodenectomy and remained free of further progression at the end of follow-up. Exocrine function was assessed in 26 patients; 11 had transient insufficiency and all recovered. No clinically recognized postoperative diabetes occurred (Table 4).

**Table 4:** Long-term oncologic and pancreatic functional outcomes.

Variable	Value
Mean follow-up, months	34.4 ± 9.8
Recurrence	1 (3.1%)
Time to recurrence	14 months
Death	0
Exocrine function assessed by fecal elastase	26/32 (81.3%)
Transient exocrine insufficiency among assessed patients	11/26 (42.3%)
Recovery of exocrine insufficiency	11/11 (100%)
Mean time to exocrine recovery, months	6.8 (range, 2–16)
Clinically recognized postoperative diabetes	0

*Values are n (%), n/N (%), mean ± standard deviation, or mean with range.*

Exploratory analyses showed clinically relevant POPF in 2/19 patients after parenchyma-preserving procedures and 0/13 after conventional resection (p = 0.502); recurrence occurred in 1/19 and 0/13, respectively (p = 1.000). Because both fistulas and the recurrence followed enucleation, enucleation-specific analyses were also performed. POPF occurred in 2/14 patients after enucleation versus 0/18 after other procedures (p = 0.183), and recurrence occurred in 1/14 versus 0/18, respectively (p = 0.438). Recurrence occurred in 1/11 R1 resections and 0/21 R0 resections (p = 0.344) (Table 5). No clinically recognized endocrine insufficiency occurred in either surgical group. Procedure-specific exocrine outcomes could not be compared reliably because fecal-elastase testing was incomplete and not uniformly distributed across procedures.

**Table 5:** Exploratory subgroup analyses.

Comparison	Outcome	Group 1	Group 2	p value
Parenchyma-preserving vs conventional resection	Clinically relevant POPF	2/19	0/13	0.502
Parenchyma-preserving vs conventional resection	Recurrence	1/19	0/13	1.000
Enucleation vs other procedures	Clinically relevant POPF	2/14	0/18	0.183
Enucleation vs other procedures	Recurrence	1/14	0/18	0.438
R1 vs R0 resection	Recurrence	1/11	0/21	0.344

*All p values are from two-sided Fisher exact tests. Parenchyma-preserving procedures comprised enucleation and central pancreatectomy; conventional resections comprised distal pancreatectomy and pancreaticoduodenectomy. These analyses were exploratory and underpowered, and absence of statistical significance should not be interpreted as equivalence.*

## Discussion

The demographic and clinical profile of this cohort was consistent with previous pediatric reports. SPN predominantly affected girls, abdominal pain was the most frequent presentation, and tumors were distributed almost equally between the pancreatic head and body/tail [2–5,18–20]. The median tumor diameter was smaller than in several published pediatric series, which may reflect earlier use of abdominal ultrasonography and cross-sectional imaging in symptomatic children [2,3,20]. Preoperative imaging suggested SPN in most patients, although four lesions were initially interpreted as pancreatic pseudocysts. This remains an important diagnostic pitfall because hemorrhage, necrosis, and cystic degeneration may produce a predominantly cystic appearance [21,22]. A presumed pancreatic pseudocyst in a child without a convincing history of pancreatitis should therefore prompt reconsideration of SPN, particularly when imaging demonstrates a capsule or an enhancing solid component.

Complete resection remains the standard treatment, but the appropriate extent of surgery is less certain. Conventional pancreatic resection provides reliable tumor removal but may sacrifice substantial normal parenchyma, with potential lifelong consequences for growth, nutrition, and endocrine and exocrine function [1,3,6,23]. Enucleation and central pancreatectomy can limit pancreatic tissue loss, while spleen-preserving distal pancreatectomy avoids the additional consequences of splenectomy [3,7,8]. In this cohort, all procedures were performed through an open approach, and all nine spleen-preserving distal pancreatectomies used splenic-vessel preservation according to the Kimura technique. The results therefore support the feasibility of preservation-oriented open surgery but cannot be extrapolated directly to laparoscopic or robotic approaches.

The principal cost of pancreatic preservation was morbidity after pancreatic head enucleation. POPF is the most frequent complication reported after SPN surgery and is more common after parenchyma-preserving procedures than after conventional resection [1]. Wu et al. similarly found more POPF after pediatric enucleation but less endocrine insufficiency, whereas Zhou et al. reported comparable fistula rates after laparoscopic and open enucleation [3,5]. In our cohort, all early complications occurred after surgery for pancreatic head tumors, and both clinically relevant fistulas followed enucleation. Exploratory comparisons showed POPF in 2 of 19 patients after parenchyma-preserving surgery and none of 13 after conventional resection, but this difference was not statistically significant and the analysis was underpowered. Tumor proximity to the main pancreatic duct or bile duct, preoperative biliary dilatation, loss of a clear dissection plane, or suspected invasion should therefore lower the threshold for formal resection rather than routine enucleation [3,7,8]. Four patients underwent a Roux-en-Y jejunal anastomosis to the enucleation bed because of anticipated leakage risk, but the selective use of this technique prevents conclusions about its effectiveness [24,25].

The R1 resection rate of 34.4% was higher than in most pediatric series [3,26,27]. This may reflect the narrow microscopic planes inherent to a preservation-oriented strategy, particularly for pancreatic head enucleation, as well as non-standardized retrospective margin sampling. Nevertheless, the current follow-up is too short to consider R1 resection oncologically inconsequential. Recurrence occurred in 1 of 11 R1 resections and none of 21 R0 resections, but the small number of events precluded meaningful risk estimation. The only recurrence combined an R1 margin with perineural invasion and Ki-67 positivity, reinforcing the need to interpret margin status together with adverse tumor biology. Positive margins, perineural or vascular invasion, tumor rupture, metastatic disease, and increased proliferative activity have been associated with recurrence, although their independent importance in children remains uncertain [1,26–28]. Complete resection of visible metastatic disease remains reasonable when feasible [29–31], whereas routine lymphadenectomy is not supported in the absence of suspicious nodes [32].

Pancreatic functional outcomes were encouraging but should be interpreted cautiously. No clinically recognized postoperative diabetes occurred, and all documented cases of exocrine insufficiency were transient. However, endocrine surveillance relied on available clinical records rather than protocolized fasting glucose, HbA1c, or oral glucose tolerance testing, and subclinical dysfunction may have been missed. Fecal elastase was available in 26 patients, but pancreatic enzyme replacement therapy, serial growth measurements, fat-soluble vitamin status, and hepatic steatosis could not be extracted uniformly. Procedure-specific exocrine outcomes could therefore not be compared reliably. These limitations are important because preservation should ultimately be judged by durable metabolic and nutritional outcomes, not only by the amount of pancreatic tissue retained.

No universally accepted pediatric SPN surveillance protocol has been established. Registry and institutional approaches support frequent clinical and imaging assessment during the first two postoperative years, followed by annual or longer-term follow-up [18,33]. A practical program should include symptoms, physical examination, height, weight and body mass index trajectories, abdominal ultrasonography or MRI, fasting glucose and HbA1c, and assessment for exocrine insufficiency with fecal elastase when indicated [33–35]. PERT use, fat-soluble vitamin status, and hepatic steatosis should be documented in patients with nutritional or metabolic abnormalities. Patients with R1 margins, tumor rupture, perineural or vascular invasion, elevated Ki-67, metastatic disease, or previous recurrence warrant closer and prolonged surveillance. Follow-up should also continue through adolescence, with planned transition to adult pancreatic, gastroenterology, nutrition, and endocrine services. Recurrent pain or pancreatitis should prompt evaluation for ductal obstruction, anastomotic stricture, anatomic variants, or genetic susceptibility [36]. Ho et al. reported preserved long-term growth in a pediatric pancreatic tumor cohort, including 20 patients with SPN, but detailed endocrine and exocrine data remained limited [37].

Larger single-center pediatric cohorts have been reported by Kim et al. and Wu et al. [3,6]. The contribution of the present study is therefore not absolute sample size, but its transparent evaluation of a preservation-oriented strategy in a consecutive cohort, including location-specific morbidity, a high R1 rate, recurrence after R1 enucleation, and pancreatic functional outcomes. The findings do not establish equivalence between surgical strategies or prove the safety of R1 resection. Rather, they support cautious, anatomy-based selection and identify the need for prospective multicenter studies with standardized operative criteria, pathology review, endocrine and exocrine testing, nutritional assessment, and transition follow-up. Future translational work may also examine regenerative adjuncts to preserve organ function, building on early cell-based approaches incorporated into pediatric hepatobiliary surgery, although their relevance to pancreatic tumor resection remains speculative [38].

### Limitations

This study was retrospective, single-center, and subject to confounding by indication because operative selection was based on tumor anatomy and surgeon judgment. The small number of events limited subgroup analyses and prevented reliable estimation of the effects of R1 resection or parenchyma preservation on recurrence and function. Immunohistochemistry was selective rather than systematic, and margin sampling was not prospectively standardized, which may have contributed to diagnostic uncertainty in a minority of cases and to the high reported R1 rate. Functional follow-up was incomplete: fecal elastase was unavailable in six patients, and PERT, serial anthropometry, protocolized glucose or HbA1c testing, and hepatic steatosis could not be uniformly extracted. Finally, follow-up was moderate, whereas late SPN recurrence can occur years after surgery.

## Conclusion

Parenchyma-preserving surgery was feasible in carefully selected children with pancreatic SPN, but pancreatic head enucleation carried clinically relevant fistula and margin-related concerns. The high R1 rate, a recurrence after R1 enucleation with adverse pathologic features, and incomplete protocolized functional follow-up support selective rather than routine pancreatic preservation, accompanied by structured long-term surveillance.

## Abbreviations

CT: computed tomography
DGE: delayed gastric emptying
HbA1c: glycated hemoglobin
MRI: magnetic resonance imaging
PERT: pancreatic enzyme replacement therapy
POPF: postoperative pancreatic fistula
SPN: solid pseudopapillary neoplasm
